# Bergmann's Body Size Rule Operates in Facultatively Endothermic Insects: Evidence from a Complex of Cryptic Bumblebee Species

**DOI:** 10.1371/journal.pone.0163307

**Published:** 2016-10-14

**Authors:** Jessica J. Scriven, Penelope R. Whitehorn, Dave Goulson, Matthew C. Tinsley

**Affiliations:** 1 Biological and Environmental Sciences, University of Stirling, Stirling, United Kingdom; 2 School of Life Sciences, University of Sussex, Brighton, United Kingdom; Scientific Research Centre of the Slovenian Academy of Sciences and Art, SLOVENIA

## Abstract

According to Bergmann’s rule we expect species with larger body size to inhabit locations with a cooler climate, where they may be well adapted to conserve heat and resist starvation. This rule is generally applied to endotherms. In contrast, body size in ectothermic invertebrates has been suggested to follow the reverse ecogeographic trend: these converse Bergmann’s patterns may be driven by the ecological constraints of shorter season length and lower food availability in cooler high latitude locations. Such patterns are particularly common in large insects due to their longer development times. As large and facultatively endothermic insects, bumblebees could thus be expected to follow either trend. In this investigation, we studied body size of three bumblebee species over a large spatial area and investigated whether interspecific trends in body size correspond to differences in their distribution consistent with either Bergmann’s or a converse Bergmann’s rule. We examined the body size of queens, males and workers of the *Bombus lucorum* complex of cryptic bumblebee species from across the whole of Great Britain. We found interspecific differences in body size corresponding to Bergmann’s rule: queens and males of the more northerly distributed, cool-adapted, species were largest. In contrast, the mean body size of the worker caste did not vary between the three species. These differences in body size may have evolved under selection pressures for thermoregulation or starvation resistance. We suggest that this case study in facultatively endothermic insects may help clarify the selection pressures governing Bergmann rule trends more generally.

## Introduction

The study of large-scale spatial variation in organismal traits has long been of interest to biologists, especially those studying ecology and evolution. Animal body size represents one of the most important quantitative traits as it strongly affects both physiology and fitness [1]. Several ecogeographic rules describing correlations between morphological variation and ecological features have been formulated [2]. Perhaps the best known is Bergmann’s rule, which predicts that endothermic vertebrate species inhabiting cooler climates will be larger than related species from warmer climates [3]. Bergmann originally used this rule to describe interspecific trends but it was later redefined to explain intraspecific variation [2–5]. This rule is generally applied to endothermic organisms and has been shown to hold for many species of bird and mammal [6–8]. The mechanism first proposed to explain these patterns was that in endotherms heat generation capacity increases with body volume, whereas heat loss increases with surface area; larger organisms, with relatively lower surface area, are therefore favoured in cooler environments [3,4].

Bergmann’s rule has also been demonstrated to apply to some groups of ectotherms, but not consistently, and where it does occur the mechanisms behind the trend may be different [1,9,10]. Ectotherms rely on heat from their environment to thermoregulate: large bodied organisms will absorb heat more slowly than smaller organisms, which could be a disadvantage in cooler climates. On the other hand, small animals may overheat more easily in hot environments (reviewed in [11]). Mousseau [12] suggested that ectotherms follow the converse Bergmann’s rule, whereby body size decreases at higher latitudes. This trend was first considered for intraspecific comparisons of body size and reported by Park [13] in a carabid beetle; it has since been found in many other arthropod species [1,12,14]. Such patterns appear to be mediated by season length rather than temperature: at high latitudes, seasons are shorter, reducing the time available for foraging, growth and development, and thus limiting the body size that can be attained [1,12]. More recently, both Blanckenhorn & Demont [1] and Shelomi [15] found that converse Bergmann clines are more commonly observed in larger bodied arthropods, such as Coleoptera and Orthoptera, as these species tend to have longer development times.

Bumblebees are large insects that usually exhibit an annual lifecycle with one generation per year. As large-bodied insects, this hypothesis predicts that bumblebees should exhibit converse Bergmann rule trends. However, bumblebees are also facultatively endothermic, generating considerable quantities of metabolic heat, both during active flight and when stationary [16–18]. In order to fly, bumblebees need to warm their flight muscles above the ambient temperature of the temperate regions where most species are found. This may be achieved by a combination of flight muscle contractions (shivering) while they are “uncoupled” from the wings [17,18] and substrate cycling in the flight muscles [19,20]. There is a limit to how much heat a bumblebee can produce and thus a minimum temperature at which they can fly [16,21]; larger individuals can produce more heat and also lose it more slowly due to their proportionally smaller surface area [17]. As such, the thermal explanations for Bergmann’s rule that are normally applied to endothermic vertebrates may operate in bumblebees, predicting that bumblebees should exhibit a positive association between body size and latitude. Here, we test these opposing hypotheses to better elucidate not only body size evolution in bumblebees, but also the mechanistic generality of Bergmann trends.

The widespread and economically exploited bumblebee subgenus, *Bombus sensu stricto* [22], includes a complex of cryptic species, about which relatively little is known. The *lucorum* complex comprises *B*. *(B*.*) lucorum* (Linnaeus), *B*. *(B*.*) cryptarum* (Fabricius) and *B*. *(B*.*) magnus* (Vogt). All three are morphologically indistinguishable in much of their range, which makes them extremely difficult to study in the field [23,24]. In the UK and Ireland, the *lucorum* complex species exhibit broadly overlapping distributions [22,23,25,26], with all three species found co-occurring at many locations [23,25,26]. However, recent work has found considerable differences in their ecology and distribution. *Bombus lucorum* is a very widespread, generalist species; in one study it was found at all locations surveyed across Great Britain (S1 Fig; [26]). In contrast, *B*. *cryptarum* and *B*. *magnus* exhibit a more restricted UK distribution than *B*. *lucorum* [25,26]; they are absent from much of southern and eastern England, and are more commonly found at sites with lower summer temperatures (S1 Fig; [26]).

A previous investigation of Bergmann’s rule in bumblebees found that although foraging workers of species inhabiting temperate regions were smaller than workers of species from cold climates, the largest species were found in the tropics [27]. This study focussed solely on workers, and included species from multiple genera, which also influenced body size variation. In this study, we use these three cryptic species, which are in most other ways ecologically and morphologically similar, as a more powerful test of Bergmann’s rule.

Carolan *et al*. [24] measured the thorax breadth of molecularly identified queens of the *lucorum* complex collected from four different European locations. While there was considerable size overlap among the species, they found that there were some significant interspecific differences in mean thorax breadth. However, these differences were inconsistent between the countries of collection: queens from Ireland showed significant variation in body size, whereas Danish queens did not [24]. In this study, we perform a large scale investigation into the body size variation of all three castes of the *lucorum* complex species across a broad geographic area to determine whether these reportedly highly similar species differ in size. Specifically, we aim to determine (i) whether these three species differ in body size (ii) if body size variation is consistent among castes, (iii) if geographic location or environmental temperature influences body size and (iv) whether trends in body size correspond to differences in their distribution consistent with either Bergmann’s or converse Bergmann’s rule.

## Materials and Methods

**Fig 1:**
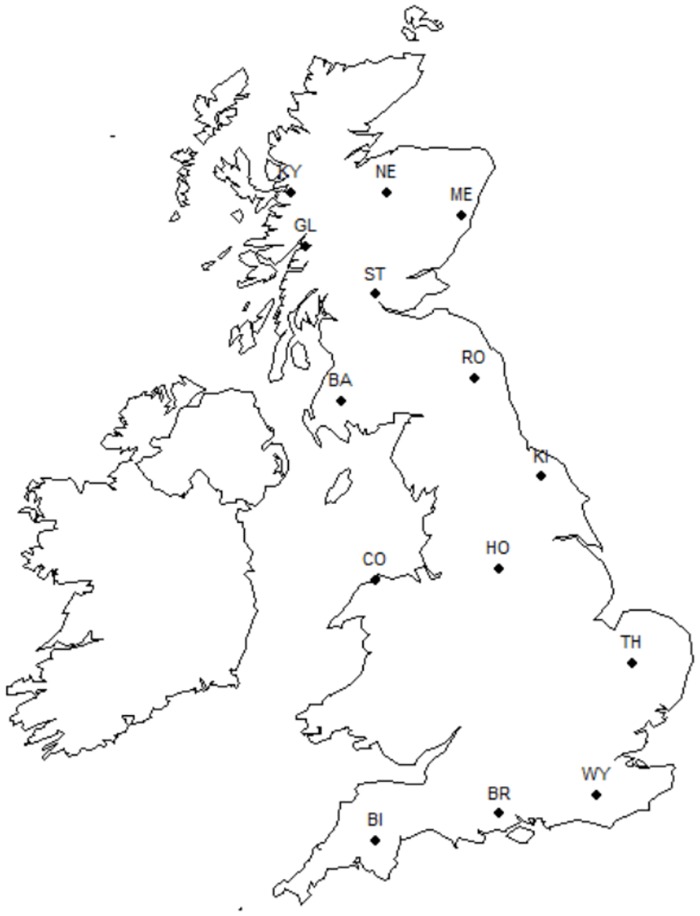
Map of collection sites for *lucorum* complex individuals.

The specimens included in this study have been used previously to assess the distribution and ecological differences of the *lucorum* complex species (see [26]). Sampling protocols were described in Scriven *et al*. [26], but only those individuals collected in 2011 were included here. In brief, workers, queens and males were sampled at 15 sites across Great Britain from June to September of 2011 (Fig 1, S1 Table). The mean number of individuals collected per site was 89.4 ± 12.9. Whole bees were stored in absolute ethanol at ambient temperatures and then identified to species level using a diagnostic mitochondrial DNA RFLP assay [25,26]. The thorax width between the tegulae (a standard measure of body size: [24]) was measured using electronic callipers.

Our statistical analysis investigated the factors influencing body size for all individuals combined and for each caste separately, fitting linear mixed effects models in R (version 3.0.2: [28]) using lmer from the lme4 package (ver. 1.1–8; [29]). Individual bee was the unit of replication, with site as a random effect. Firstly, we tested whether body size (thorax width) differed between the three species in a model including all three castes: the model contained the terms ‘species’ and ‘caste’ as well as their interaction. We then investigated whether thorax width differed among the three species for each caste separately and determined whether three site-level covariates, latitude (degrees), elevation (m) and mean daily temperature (°C) [30] from March to August (the approximate flight period for the three species), influenced body size. For this analysis we mean-centred these covariates and tested for non-linear effects by fitting second order polynomial functions. We found two queens that were sampled at each extreme of the temperature range to be highly influential in the models, so we removed these two individuals from further analyses. The data set for queens was unsuitable for effectively investigating the effect of latitude, elevation and temperature, due to smaller sample sizes and an uneven distribution across sites; therefore we did not include these variables in models of queen size variation.

We investigated whether the pattern of interspecific body size differences varied among sites by testing for a site by species random effect interaction. The significance of fixed effects and their interactions was tested using likelihood ratio tests to compare models with and without the term of interest. For random effect terms we assessed model fit using AICc values. Pairwise differences between factor means were investigated using Tukey’s post hoc tests.

## Results

**Fig 2:**
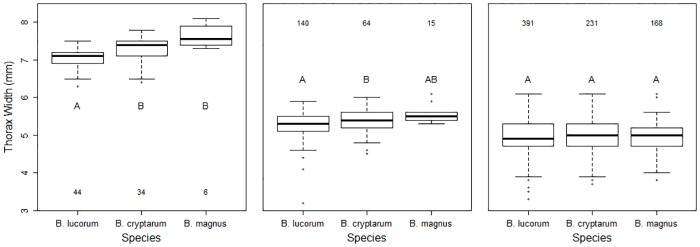
Differences in body size of the three bumblebee species. The thorax widths of (a) queens, (b) males and (c) workers of *B*. *lucorum*, *B*. *magnus* and *B*. *cryptarum*. Box and whisker plots compare medians. Numbers give sample sizes. Different letters denote categories for which the means are significantly different (*P* < 0.01). The plots are based on raw data.

Thorax width was recorded for 1,095 bees belonging to the lucorum complex species, which comprised 575 B. lucorum, 330 B. cryptarum and 190 B. magnus individuals (S1 Table). These three cryptic species showed caste-specific differences in their mean body size (Fig 2): a significant interaction between species and caste (Table 1) demonstrated that the interspecific differences in thorax width were not consistent across the three castes. Whilst the three species exhibited significantly different thorax widths for both queens and males (Table 2; Fig 2a and 2b), for workers there was no significant interspecific variation (Table 2; Fig 2c). Posthoc tests revealed that both B. cryptarum and B. magnus queens were significantly larger than B. lucorum queens (Fig 2a): the mean thorax breadths were 3.3% larger in B. cryptarum (t = 3.2, df = 80.8, P = 0.005) and 9.6% larger in B. magnus (t = 3.2, df = 21.4, P = 0.01). The mean size of B. magnus males was larger than either of the other species (6.1% bigger than B. lucorum and 3.3% bigger than B. cryptarum); however pairwise post hoc tests for B. magnus compared to B. lucorum and B. cryptarum were not significant (t = 2.3, df = 12.6, P = 0.09 and t = 1.2, df = 13.6, P = 0.48 respectively; Fig 2b). Nevertheless, B. cryptarum males were significantly larger than B. lucorum males (t = 3, df = 216, P = 0.008; Fig 2b), which represented a 2.7% difference in mean thorax width. There were no significant differences between the body size of B. cryptarum and B. magnus for any of the castes.

**Table 1:** Caste-specific interspecific differences in thorax width across the *lucorum* complex species.

Fixed effects		Estimate	SE	χ^2^	*P*
Intercept (*B*. *cryptarum* males)		5.4	0.06		
Caste				1284.2	*<0*.*001*
	Queens	2.02	0.08		
	Workers	-0.44	0.05		
Species				4.9	0.085
	*B*. *lucorum*	-0.15	0.06		
	*B*. *magnus*	0.12	0.15		
Caste:Species				13.4	*0*.*009*
	Queens: *B*. *lucorum*	-0.04	0.1		
	Workers: *B*. *lucorum*	0.15	0.07		
	Queens: *B*. *magnus*	-0.05	0.22		
	Workers: *B*. *magnus*	-0.18	0.16		
**Random effect variance**					
Site		0.01			
Residual		0.14			

The size differences (thorax width in mm) between the three *lucorum* complex species. Summary of the results of linear mixed effects models. *B*. *cryptarum* was the reference (intercept) species, parameter estimates for other species are given as contrasts relative to this. Significant results are shown in italics.

**Table 2:** Differential interspecific variation in thorax width among the three bumblebee castes.

		Queens				Males				Workers			
Covariates		Estimate	SE	χ^2^	*P*	Estimate	SE	χ^2^	*P*	Estimate	SE	χ^2^	*P*
*Intercept (B*. *lucorum)*		6.97	0.11			5.26	0.04			4.97	0.04		
Species				20.5	*<0*.*001*			13.81	*0*.*001*			2.08	0.35
	*B*. *cryptarum*	0.23	0.07			0.14	0.05			0	0.04		
	*B*. *magnus*	0.67	0.17			0.32	0.13			-0.06	0.05		
**Random effect variance**													
Site		0.02				0.01				0.02			
Residual		0.09				0.09				0.16			

The size differences (thorax width in mm) between the three *lucorum* complex species for queens, males and workers. Summary of the results of linear mixed effects models. *B*. *lucorum* was the reference (intercept) species, parameter estimates for other species are given as contrasts relative to this. Significant results (testing for variation between all three species) are shown in italics.

Our analysis provided no support for an interaction between site and species, meaning that the interspecific variation in body size was consistent across sites: including a species by site random effect interaction did not improve the model (model with all three castes: AICc = 1034.3 with interaction, and AICc = 1029.5 without). We did not detect a significant effect of mean summer temperature, latitude or altitude on body size; however, the non-significant trend that existed for workers suggested that they became larger at higher latitudes (S2 Table. There was also no evidence of interactions between species and mean summer temperature, latitude or altitude; meaning that there was no variation across the three species in how these covariates were associated with body size. Lastly, there was no evidence for non-linear relationships for any continuous predictors.

## Discussion

In this study we find caste-specific body size differences between the three member species of the *lucorum* complex of cryptic bumblebees, which have previously been described as near-morphologically identical [23,24,31]. Of the three species, *B*. *magnus* and *B*. *cryptarum*, which predominantly inhabit cooler and more northerly locations in the UK, had queens and males that were significantly larger than those of *B*. *lucorum*.

Bergmann’s rule was originally posited to explain geographic variation in body size of endothermic vertebrates [3]. There has been considerable debate about how it should be applied to insects and other invertebrates, some authors suggesting that ectothermic insects with large body size should exhibit converse Bergmann clines [1,12]. Our study questioned which pattern bumblebees should adhere to, as they are both large and, unusually for insects, facultatively endothermic. In this study of multiple populations of three cryptic bumblebee species, we find evidence for interspecific variation in body size that corresponds to Bergmann’s rule.

In Great Britain, *B*. *cryptarum* and *B*. *magnus* occur more commonly where temperatures are lower, and are more abundant at northerly latitudes, a pattern that is not evident for *B*. *lucorum* [26]. They are also more active than *B*. *lucorum* when conditions are cooler and cloudier [32]. Similarly, in Austria mean annual air temperature was lower for sampling sites where *B*. *cryptarum* was found, than for sites with *B*. *lucorum*; additionally *B*. *cryptarum* was relatively more common at higher altitudes [33]. This growing body of evidence suggests that *B*. *lucorum* is adapted for activity in warmer conditions than *B*. *magnus* or *B*. *cryptarum*. Our present results, demonstrating larger mean body size of *B*. *cryptarum* and *B*. *magnus* reproductives (queens and males) compared to *B*. *lucorum*, are consistent with the theory that there is divergent thermal specialisation between the species of the *lucorum* complex [32].

The foremost hypotheses to explain species distribution patterns in agreement with Bergmann’s rule are the heat conservation hypothesis and the starvation resistance hypothesis [6]. Both could explain why only the reproductive castes display Bergmann body size differences in the *lucorum* complex species. Larger bumblebees generate more heat [16,17] and large body size creates a lower surface area to volume ratio that reduces heat loss. In the UK, queen and male bumblebees of these species are on the wing early (spring) and late (autumn) in the flight season, when conditions are likely to be coldest [21,32,34]. Thus, it is these castes that would benefit most from enhanced heat conservation in species with northerly distributions. Regarding starvation resistance, the greater likelihood of bad weather at times when these castes are active may often prevent queens and males from foraging. Moreover, mated queens spend the winter in diapause, surviving only on the fat reserves that fill their abdominal cavity [21,34]. Small queens are less likely to survive this diapause period [35], which is longer in the north and west of the UK [36], meaning larger queens are better equipped to resist starvation. Selection may thus be strongest on the queens and males, which gain the highest fitness benefits from large body size in the more northerly distributed species, *B*. *magnus* and *B*. *cryptarum*. Furthermore, any differences among body size in workers may be masked by the high variation in worker body size for all three species. This is a common feature of bumblebees, where workers can vary up to ten-fold both within species and even with a single colony [34,37].

Despite the propensity for large insects to display converse Bergmann trends [1], our data suggest that bumblebees do not. One hypothesis to explain the existence of converse Bergmann trends is that body size is limited in cooler habitats by short growing seasons [1,12]. We suggest that the combination of facultative endothermy and colonial eusociality in bumblebees, which means offspring are reared in a warmed nest environment by numerous individuals [17,21], may ameliorate the short growing season constraints on body size that result in converse Bergmann trends in other large invertebrates.

This study detects interspecific body size differences in accordance with Bergmann’s rule but does not find strong evidence for similar intraspecific trends. Bergmann’s ecogeographic rule has been modified to describe intraspecific variation in body size [2,4,6] but it was originally formulated to explain size differences between taxa [3,5,6,8]. Since then, both intraspecific [1,5,9,12] and interspecific [11,38–40] Bergmann’s gradients and their converse have been observed. Although not significant in our study, model parameter estimates suggested a trend that workers of all three species tend to be larger at higher latitudes (corresponding to an approximate 5% size increase across the length of the UK); this is consistent with Bergmann’s rule and would be worthy of future investigation.

All three of these bumblebee species have large global distributions [22], so although this study included a broad geographic area, it still only represents a fraction of their total range. Expanding the study to encompass a larger area might therefore reveal stronger intraspecific body size trends. Currently the best data on the worldwide distribution of these three species has been provided by Williams *et al*. [22] but samples were limited, particularly for *B*. *magnus*. Based on the findings here, it might be expected that the distributions of *B*. *cryptarum* and *B*. *magnus* extend further northwards or to higher elevations than that of *B*. *lucorum*, but additional work would be required to confirm this.

The extent to which overlooked interspecific morphological variation exists within cryptic species complexes has received considerable interest [41–44]. Here we show that, despite significant body size distribution-overlap among the bumblebee species of the *lucorum* complex, these cryptic species have diverged significantly in the mean body size of their reproductive castes. Queens and males of *B*. *cryptarum* and *B*. *magnus* were larger than those of *B*. *lucorum*, whereas there were no interspecific body size differences in workers. This strongly suggests that divergent caste-specific selection pressures have acted on body size in these species.

## Conclusions

Bergmann’s rule is a classic example of adaptive geographic variation [45,46], nevertheless, the extent to which Bergmann’s rule applies is still debated, particularly in the case of ectotherms [1,6–9,15]. In this study we find evidence for interspecific body size variation between three closely related cryptic bumblebee species consistent with Bergmann’s rule. We propose that these interspecific size differences may have been driven by selection pressures on thermoregulation and starvation resistance. Furthermore, bumblebees are an exception amongst invertebrates because they are facultatively endothermic; this case study may therefore help clarify the selection pressures that climate exerts on body size, providing indirect confirmation of the underlying explanation for the existence of Bergmann trends in other ectotherms.

## Supporting Information

S1 Fig The distribution of the three *lucorum* complex species across Great Britain. Sites marked with a * were sampled in 2011, those without were sampled in 2010. Taken from Scriven et al. (2015).
(DOCX)

S1 Table Sample sizes from each of the sites across Great Britain following species determination. Summary of the data collected for each sampling site along with the numbers of each of the three *lucorum* complex species caught at each site. Temperature was measured as the mean daily temperature from March-August, which is the approximate flight period of these species.
(DOCX)

S2 Table The size differences (thorax width in mm) between the three *lucorum* complex species and three site-level covariates, latitude (°), altitude (m) and mean daily temperature (°C), for queens, males and workers. Summary of the results of the full linear mixed effects models before model simplification. *Bombus lucorum* was the reference (intercept) species, parameter estimates are given as contrasts relative to this. Significant results are shown in italics. The data set for queens was unsuitable for effectively investigating the effect of latitude, altitude and temperature (see text); therefore we did not include these variables in models of queen size variation.
(DOCX)
