# Supplementary figures and images for: Bergmann's Body Size Rule Operates in Facultatively Endothermic Insects: Evidence from a Complex of Cryptic Bumblebee Species
Source: PLoS One. 2016 Oct 14;11(10):e0163307. doi: 10.1371/journal.pone.0163307 (PMC5065188; doi:10.1371/journal.pone.0163307)

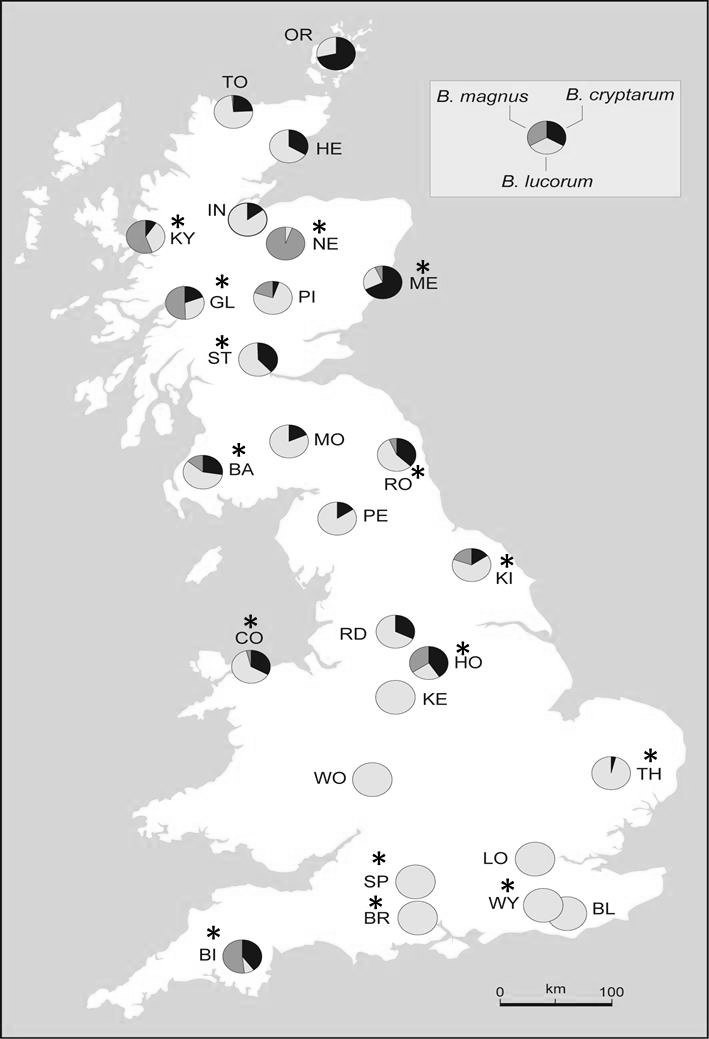


**S1 Fig**

Supplement: S1 Fig — Sites marked with a * were sampled in 2011, those without were sampled in 2010. Taken from Scriven et al. (2015). (DOCX) [file pone.0163307.s001.docx]
