# Supplementary material for: Bergmann's Body Size Rule Operates in Facultatively Endothermic Insects: Evidence from a Complex of Cryptic Bumblebee Species
Source: PLoS One. 2016 Oct 14;11(10):e0163307. doi: 10.1371/journal.pone.0163307 (PMC5065188; doi:10.1371/journal.pone.0163307)
Supplement: S1 Table — Summary of the data collected for each sampling site along with the numbers of each of the three lucorum complex species caught at each site. Temperature was measured as the mean daily temperature from March-August, which is the approximate flight period of these species. (DOCX) [file pone.0163307.s002.docx]

| **Site** | **Habitat** | **Latitude** | **Longitude** | **Altitude (m)** | **Temp. (°C)** | ***B. cryptarum*** | ***B. lucorum*** | ***B. magnus*** | **Total** |
| --- | --- | --- | --- | --- | --- | --- | --- | --- | --- |
| Kyle of Lochalsh (KY) | Heathland | 57.23 | -5.40 | 15 | 9.0 | 1 | 7 | 42 | 50 |
|  | Non-heathland | 57.28 | -5.52 | 10 | 11.1 | 6 | 20 | 0 | 26 |
| Nethy Bridge (NE) | Heathland | 57.23 | -3.68 | 260 | 9.9 | 0 | 1 | 16 | 17 |
| Mergie (ME) | Heathland | 57.00 | -2.34 | 165 | 9.3 | 52 | 6 | 5 | 63 |
|  | Non-heathland | 56.99 | -2.29 | 130 | 9.6 | 22 | 22 | 2 | 46 |
| Glencoe (GL) | Heathland | 56.66 | -5.05 | 85 | 10.4 | 2 | 2 | 51 | 55 |
|  | Non-heathland | 56.68 | -5.12 | 15 | 9.9 | 18 | 29 | 1 | 48 |
| Stirling (ST) | Heathland | 56.19 | -3.89 | 318 | 9.6 | 87 | 47 | 0 | 134 |
|  | Non-heathland | 56.14 | -3.92 | 50 | 10.3 | 15 | 87 | 0 | 102 |
| Rothbury (RO) | Heathland | 55.34 | -2.12 | 150 | 9.4 | 25 | 15 | 6 | 46 |
|  | Non-heathland | 55.29 | -1.85 | 105 | 10.6 | 11 | 39 | 0 | 50 |
| Bargrennan (BA) | Heathland | 55.11 | -4.49 | 270 | 9.3 | 4 | 1 | 7 | 12 |
|  | Non-heathland | 55.01 | -4.54 | 40 | 11.0 | 10 | 28 | 0 | 38 |
| Kirkbymoorside (KI) | Heathland | 54.33 | -0.94 | 215 | 10.6 | 10 | 28 | 15 | 53 |
|  | Non-heathland | 54.22 | -0.88 | 30 | 11.7 | 2 | 23 | 0 | 25 |
| Hope (HO) | Heathland | 53.39 | -1.69 | 340 | 10.6 | 21 | 3 | 25 | 49 |
|  | Non-heathland | 53.35 | -1.75 | 170 | 11.6 | 9 | 15 | 0 | 24 |
| Conwy (CO) | Heathland | 53.28 | -3.88 | 190 | 12.6 | 11 | 26 | 2 | 39 |
|  | Non-heathland | 53.23 | -3.84 | 25 | 12.0 | 7 | 8 | 0 | 15 |
| Thetford (TH) | Heathland | 52.42 | 0.71 | 45 | 12.4 | 3 | 36 | 0 | 39 |
|  | Non-heathland | 52.40 | 0.92 | 40 | 12.6 | 0 | 34 | 0 | 34 |
| Wych Cross (WY) | Heathland | 51.07 | 0.05 | 140 | 12.0 | 0 | 34 | 0 | 34 |
| Bramshaw (BR) | Heathland | 50.89 | -1.69 | 100 | 12.6 | 0 | 49 | 0 | 49 |
|  | Non-heathland | 50.95 | -1.78 | 50 | 12.7 | 0 | 12 | 0 | 12 |
| Birch Tor (BI) | Heathland | 50.61 | -3.87 | 421 | 10.5 | 14 | 3 | 18 | 35 |

**S1 Table**
