# Supplementary material for: Bergmann's Body Size Rule Operates in Facultatively Endothermic Insects: Evidence from a Complex of Cryptic Bumblebee Species
Source: PLoS One. 2016 Oct 14;11(10):e0163307. doi: 10.1371/journal.pone.0163307 (PMC5065188; doi:10.1371/journal.pone.0163307)
Supplement: S2 Table — Summary of the results of the full linear mixed effects models before model simplification. Bombus lucorum was the reference (intercept) species, parameter estimates are given as contrasts relative to this. Significant results are shown in italics. The data set for queens was unsuitable for effectively investigating the effect of latitude, altitude and temperature (see text); therefore we did not include these variables in models of queen size variation. (DOCX) [file pone.0163307.s003.docx]

**S2 Table**

|  | **Queens** | | | |  | **Males** | | | |  | **Workers** | | | |
| --- | --- | --- | --- | --- | --- | --- | --- | --- | --- | --- | --- | --- | --- | --- |
| **Fixed effects** | **Estimate** | **SE** | **χ^2^** | ***P*** |  | **Estimate** | **SE** | **χ^2^** | ***P*** |  | **Estimate** | **SE** | **χ^2^** | ***P*** |
| *B. lucorum* | 6.97 | 0.09 |  |  |  | 5.27 | 0.05 |  |  |  | 4.97 | 0.05 |  |  |
| Species (*B. cryptarum*) | 0.27 | 0.08 | 20.50 | *<0.001* |  | 0.13 | 0.05 | 9.07 | *0.01* |  | 0.00 | 0.04 | 1.26 | 0.53 |
| Species (*B. magnus*) | 0.65 | 0.16 |  |  |  | 0.30 | 0.16 |  |  |  | -0.06 | 0.05 |  |  |
| Latitude | NA | NA | NA | NA |  | 0.001 | 0.04 | 0.05 | 0.83 |  | 0.03 | 0.02 | 2.54 | 0.11 |
| Elevation | NA | NA | NA | NA |  | 0.00 | 0.00 | 0.05 | 0.82 |  | 0.00 | 0.00 | 0.36 | 0.54 |
| Mean temperature | NA | NA | NA | NA |  | -0.03 | 0.07 | 0.03 | 0.56 |  | 0.04 | 0.04 | 1.32 | 0.25 |
|  |  |  |  |  |  |  |  |  |  |  |  |  |  |  |
| **Random effect variance** |  |  |  |  |  |  |  |  |  |  |  |  |  |  |
| Site | 0.02 |  |  |  |  | 0.02 |  |  |  |  | 0.02 |  |  |  |
| Residual | 0.09 |  |  |  |  | 0.09 |  |  |  |  | 0.16 |  |  |  |
